# Prediction of Cardiogenic Shock in Acute Myocardial Infarction Patients Using a Nomogram

**DOI:** 10.3390/jcm14248789

**Published:** 2025-12-12

**Authors:** Jie Wang, Changying Zhao, Chuqing Yang, Yang Dong, Xiaohong Yang, Chaofeng Sun

**Affiliations:** 1 Department of Hematology, The First Affiliated Hospital of Xi’an Jiaotong University, No. 277 Yanta West Road, Xi’an 710061, China; 2 Department of Cardiovascular Surgery, The First Affiliated Hospital of Xi’an Jiaotong University, No. 277 Yanta West Road, Xi’an 710061, China; 3 Health Science Center, Xi’an Jiaotong University, No. 76 Yanta West Road, Xi’an 710061, China; 4 Department of Cardiovascular Medicine, The First Affiliated Hospital of Xi’an Jiaotong University, No. 277 Yanta West Road, Xi’an 710061, China

**Keywords:** acute myocardial infarction, cardiogenic shock, case–control study, prediction model

## Abstract

**Background**: Cardiogenic shock (CS) complicating acute myocardial infarction (AMI) is associated with a high mortality rate. Early identification of patients at risk for in-hospital CS is crucial for timely intervention. This study aimed to develop a risk prediction model for CS using admission data. **Methods**: This retrospective case–control study included AMI patients and classified them into case and control groups, based on the development of in-hospital CS. Clinical information at admission was obtained and 1:1 propensity score matching (PSM) was performed based on age, gender, and diagnosis of ST-elevation myocardial infarction. Factors with *p* < 0.10 at baseline were incorporated to identify the independent risk factors, which were further used to construct a predictive nomogram. **Results**: After PSM, 374 patients were finally enrolled in both groups. After relaxed least absolute shrinkage and selection operator and multivariate logistic regression, independent risk factors identified for CS in AMI patients included systolic blood pressure [odds ratio (OR): 0.866; 95% confidence interval (CI): 0.844–0.888, *p* < 0.001], diastolic blood pressure (OR: 1.031; 95% CI: 1.001–1.063, *p* = 0.046), triglycerides (OR: 0.561; 95% CI: 0.385–0.820, *p* = 0.003), creatinine (OR: 1.005; 95% CI: 1.000–1.010, *p* = 0.048), globulin (OR: 0.915; 95% CI: 0.862–0.972, *p* = 0.004), left ventricular ejection fraction (OR: 0.951; 95% CI: 0.928–0.975, *p* < 0.001), and coronary angiography (OR: 0.183; 95% CI: 0.058–0574, *p* = 0.004). The nomogram incorporating these variables demonstrated an area under the curve of 0.937 (95% CI: 0.952–0.967), indicating good discriminatory ability in the calibration curve and decision curve. **Conclusions**: Seven independent risk factors for CS in AMI patients were identified upon admission. The proposed nomogram might facilitate early risk stratification and guide clinical decision-making to improve outcomes.

## 1. Introduction

Acute myocardial infarction (AMI) is a life-threatening cardiac emergency caused by abrupt coronary artery occlusion, resulting in myocardial ischemia and necrosis [1]. It is a leading cause of sudden cardiac death, characterized by rapid onset, high incidence and poor prognosis [2,3]. In the United States alone, the total annual economic burden of AMI was estimated at USD 84.9 billion in 2016, comprising USD 29.8 billion in direct medical costs and USD 55.7 billion in lost productivity [4]. These statistics underscore the substantial global impact of AMI on both population health and healthcare systems.

Cardiogenic shock (CS) is a life-threatening condition of persistent hypoperfusion due to cardiac pump failure, leading to end-organ dysfunction and hyperlactatemia [5,6]. Recent epidemiological data indicate that about 30% of CS cases in contemporary cardiac intensive care units are still attributable to AMI [7]. Despite advances in early revascularization, optimal medical therapy, and mechanical circulatory support, the survival rates among AMI patients with CS remain poor [8]. The Society for Cardiovascular Angiography and Intervention has proposed a clinical staging classification for CS, which identifies distinct mortality risk levels—ranging from 7.1% in stage B (beginning) to 67.0% in stage E (extremis) [9]. Notably, even patients classified as stage B (at-risk) exhibit a significantly elevated risk of mortality, underscoring the importance of identifying individuals at the earliest signs of compromise.

In conclusion, early identification and prevention of CS in high-risk AMI patients are crucial for improving clinical outcomes. This study aimed to identify independent risk factors at admission and to construct a predictive model to provide support for the early prediction of in-hospitalization CS.

## 2. Materials and Methods

### 2.1. Study Design

This retrospective case–control study included patients who were diagnosed with AMI and received treatment at the First Affiliated Hospital of Xi’an Jiaotong University between January 2018 and December 2022. The diagnosis of AMI was based on the universal definition criteria set forth by the American College of Cardiology [10]. The exclusion criteria were as follows: (1) patients younger than 18 years or older than 80 years of age; (2) presence of other comorbidities that may significantly affect prognosis; (3) patients diagnosed with CS at the time of admission; (4) incomplete clinical data.

This study was approved by the Ethics Committee of the First Affiliated Hospital of Xi’an Jiaotong University (No. XJTU1AF2024LSYY-451, Approval Date: 27 November 2024) and was conducted in accordance with the Declaration of Helsinki. The Ethics Committee of the First Affiliated Hospital of Xi’an Jiaotong University.

### 2.2. Grouping and Data Collection

Demographic characteristics, biochemical and imaging results, and other relevant clinical information at admission of all patients were collected from the electronic medical records in the Biobank of the First Affiliated Hospital of Xi’an Jiaotong University.

**Figure 1:**
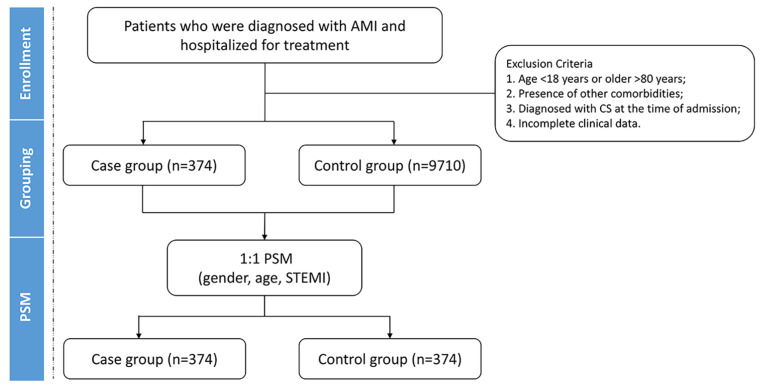
Flowchart of this case–control study. AMI, acute myocardial infarction; CS, cardiogenic shock; PSM, propensity score matching; STEMI, ST-elevation myocardial infarction.

AMI patients who experienced CS during hospitalization were categorized into the case group, and others were categorized into the control group. The diagnosis of CS was defined as sustained systolic arterial pressure < 90 mmHg for ≥30 min (or the need for vasopressor support to maintain a mean arterial pressure ≥ 65 mmHg), accompanied by signs of organ hypoperfusion, such as altered mental status, cold extremities, oliguria, or elevated lactate levels [11]. All patients were classified as Society for Cardiovascular Angiography and Interventions Stage B (beginning of shock) or higher, consistent with established criteria [12]. A 1:1 propensity score matching (PSM) was performed based on age, gender, and diagnosis of ST-elevation myocardial infarction, with a caliper value set at 0.05 (Figure 1).

### 2.3. Statistical Analysis

This was a retrospective case–control study. For variables with missing values, multiple imputation was employed to generate complete datasets. Continuous variables that adhere to a normal distribution were presented as mean with standard deviation and compared using Student’s *t*-test. For other continuous variables, medians along with interquartile ranges were reported, and comparisons were made using Mann–Whitney U-test. Categorical variables were described in terms of counts and percentages, with comparisons conducted using either chi-square test or Fisher’s exact test. Univariate and multivariate logistic regression analyses were performed to identify the risk factors. Variables showing *p* < 0.10 in baseline analyses were incorporated into relaxed least absolute shrinkage and selection operator (LASSO) logistic regression and multivariate logistic regression analysis to identify independent risk factors. Odds ratios (ORs) and 95% confidence intervals (CIs) were calculated to determine the strength of risk factors. Independent risk factors were used to construct a nomogram. Model discrimination was assessed using the receiver operating characteristic curve, with the area under the curve (AUC) used to quantify predictive performance. Calibration analysis was performed to evaluate the agreement between predicted probabilities and observed outcomes, with results presented graphically using a calibration plot. To assess clinical usefulness, decision curve analysis was conducted to determine whether the nomogram provides a net benefit across a range of clinically relevant threshold probabilities. IBM SPSS Statistics (version 27.0) and R software (version 4.5.1) were used for statistical analysis. A two-sided *p*-value < 0.05 was considered statistically significant.

## 3. Results

A total of 10,084 patients diagnosed with AMI were included in this retrospective analysis. Among them, 374 patients (3.71%) developed CS during hospitalization and were assigned to the case group. Before PSM, significant differences were observed between groups in terms of gender, age, and prevalence of ST-elevation myocardial infarction (all *p* < 0.001). After PSM, the proportion of male patients [272 (72.7) vs. 274 (73.3), *p* = 0.869], age [(63 ± 12) vs. (63 ± 11), *p* = 0.954], and diagnosis of ST-elevation myocardial infarction [263 (70.3) vs. 264 (70.6), *p* = 0.936] showed no statistically significant differences between the groups (Table 1).

### 3.1. Baseline Characteristics

The demographic characteristics and biochemical parameters of the two groups are presented in Table 2. Overall, the case group had a lower body mass index [23.73 ± 3.23 vs. 24.52 ± 2.99, *p* = 0.001] and lower prevalence of hypertension [141 (37.7) vs. 203 (54.3), *p* < 0.001] and diabetes [102 (27.3) vs. 130 (34.8), *p* = 0.027]. Compared with the control group, the case group exhibited higher levels of white blood cells [10.47 (7.80, 13.16) vs. 8.76 (6.82, 11.16), *p* < 0.001], neutrophils [8.34 (5.60, 11.31) vs. 6.56 (4.81, 9.22), *p* < 0.001], neutrophil percentage [81.05 (72.90, 86.70) vs. 77.10 (69.05, 84.33), *p* < 0.001], c-reactive protein [58.78 (31.90, 85.84) vs. 49.63 (21.43, 83.46), *p* = 0.036], lactate dehydrogenase [350.00 (240.50, 655.75) vs. 292.50 (228.00, 439.25), *p* < 0.001], creatine kinase [587.14 (145.50, 1460.00) vs. 301.00 (107.75, 901.00), *p* < 0.001], creatine kinase MB isoenzyme [63.40 (20.04, 176.63) vs. 33.00 (16.00, 109.93), *p* < 0.001], n-terminal pro-brain natriuretic peptide [1372.50 (330.25, 3553.65) vs. 813.10 (315.18, 2295.75), *p* = 0.002], urea [6.16 (4.94, 7.85) vs. 5.57 (4.55, 7.10), *p* < 0.001], creatinine [72.00 (57.00, 96.00) vs. 64.50 (53.00, 79.25), *p* < 0.001], aspartate transaminase [84.00 (32.00, 240.00) vs. 49.00 (27.00, 124.50), *p* < 0.001], alanine transaminase [41.50 (24.75, 72.50) vs. 32.00 (21.00, 58.50), *p* < 0.001], D-dimer [0.83 (0.46, 2.32) vs. 0.55 (0.33, 1.01), *p* < 0.001], and fibrin degradation product [2.50 (1.50, 7.55) vs. 2.00 (1.20, 3.60), *p* < 0.001]. Meanwhile, the case group exhibited lower levels of systolic blood pressure (SBP) [89 (84, 90) vs. 126 (111, 140), *p* < 0.001], diastolic blood pressure (DBP) [59 (54, 65) vs. 77 (68, 88), *p* < 0.001], hemoglobin [134 (120, 147) vs. 139 (126, 150), *p* = 0.001], lymphocytes percentage [13.45 (8.85, 19.89) vs. 15.70 (10.06, 23.52), *p* = 0.002], HbA1c [5.80 (5.50, 6.50) vs. 6.10 (5.60, 7.50), *p* < 0.001], triglycerides (TG) [1.08 (0.73, 1.53) vs. 1.27 (0.90, 1.80), *p* < 0.001], globulin [24.76 ± 4.47 vs. 26.10 ± 4.08, *p* < 0.001], albumin [36.30 ± 5.12 vs. 37.65 ± 4.91, *p* < 0.001], and left ventricular ejection fraction (LVEF) [49 ± 11 vs. 54 ± 11, *p* < 0.001]. Additionally, the case group exhibited a lower proportion of coronary angiography (CAG) [330 (88.2) vs. 357 (95.5), *p* < 0.001] procedures, longer in-hospital stays [5 (3, 7) vs. 4 (2, 6), *p* = 0.009], and higher in-hospital mortality [20 (5.3) vs. 6 (1.6), *p* = 0.005]. There was no significant difference between the two groups in terms of other characteristics.

### 3.2. LASSO-Logistic Regression and Multivariate Logistic Regression Analysis

**Figure 2:**
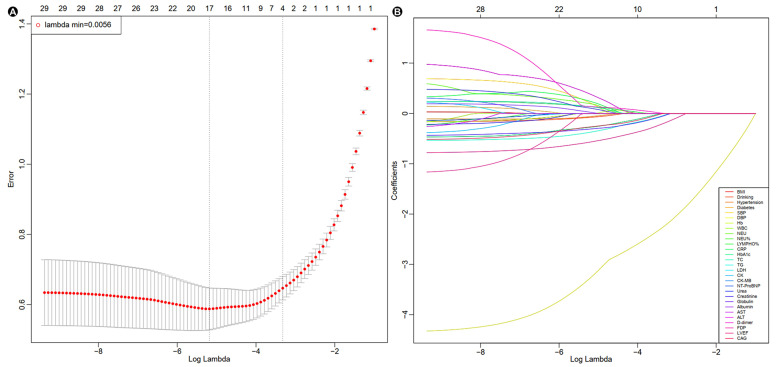
LASSO-logistic regression results. (**A**) Cross-validation plot. (**B**) Selection process by cross-validation method. BMI, body mass index; SBP, systolic blood pressure; DBP, diastolic blood pressure; Hb, hemoglobin; WBC, white blood cells; NEU, neutrophils; NEU%, neutrophil percentage; LYMPHO%, lymphocytes percentage; CRP, C-reactive protein; TC, total cholesterol; TG, triglycerides; LDH, lactate dehydrogenase; CK, creatine kinase; CK-MB, creatine kinase-MB isoenzyme; NT-proBNP, N-terminal pro-brain natriuretic peptide; AST, aspartate aminotransferase; ALT, alanine aminotransferase; FDP, fibrin degradation products; LVEF, left ventricular ejection fraction; CAG, coronary angiography.

These variables were further evaluated by LASSO logistic regression. When λ-min = 0.0056, seventeen independent risk factors were identified (Figure 2 and Table 3). After multivariate logistic regression analysis, SBP (OR: 0.866; 95% CI: 0.844–0.888, *p* < 0.001), DBP (OR: 1.031; 95% CI: 1.001–1.063, *p* = 0.046), TG (OR: 0.561; 95% CI: 0.385–0.820, *p* = 0.003), creatinine (OR: 1.005; 95% CI: 1.000–1.010, *p* = 0.048), globulin (OR: 0.915; 95% CI: 0.862–0.972, *p* = 0.004), LVEF (OR: 0.951; 95% CI: 0.928–0.975, *p* < 0.001), and CAG (OR: 0.183; 95% CI: 0.058–0.574, *p* = 0.004) were identified as independent risk factors for CS in AMI patients (Table 3).

### 3.3. Construction of Nomogram

**Figure 3:**
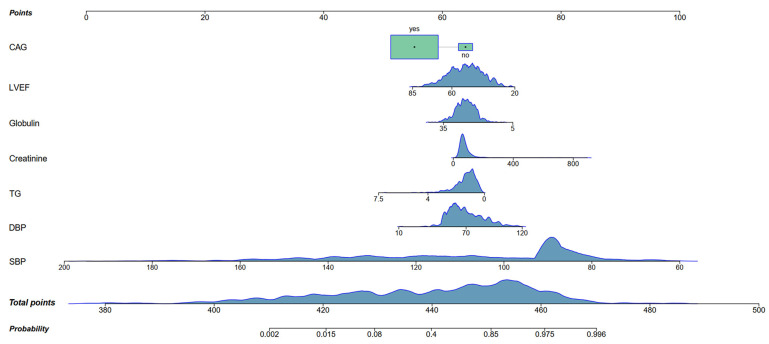
Nomogram to predict the probability of in-hospital CS for AMI patients. SBP, systolic blood pressure; DBP, diastolic blood pressure; TG, triglycerides; LVEF, left ventricular ejection fraction; CAG, coronary angiography.

**Figure 4:**
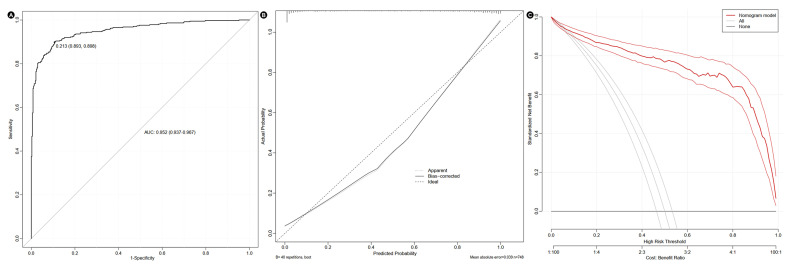
ROC curve (**A**), calibration curve (**B**), and decision curve (**C**) of the nomogram. AUC, areas under the curve.

A nomogram was constructed to predict the incidence of CS in AMI patients based on all independent risk factors (Figure 3). Based on admission data and situation, every patient can take a single point for SBP, DBP, TG, creatinine, globulin, LVEF, and CAG. The sum of these seven components yielded a total point that correlated with the predicted probability of developing in-hospital CS. The predictive model exhibited good discriminatory power, as evidenced by an AUC of 0.937 (95% CI: 0.952–0.967), indicating a high ability to distinguish between patients who would develop CS and those who would not. Furthermore, the calibration curve demonstrated close agreement between predicted and observed probabilities, suggesting that the model’s estimations were well-aligned with actual clinical outcomes. In addition, decision curve analysis revealed a favorable net clinical benefit across a wide range of threshold probabilities, supporting the model’s practical utility in guiding clinical decision-making (Figure 4).

## 4. Discussion

This study included a total of 10,084 AMI patients, consisting of 374 (3.71) in the case group. Patients were assigned to either the case or control group based on their diagnosis of CS during the hospitalization. A 1:1 PSM was performed based on age, gender, and diagnosis of ST-elevation myocardial infarction, with a caliper value set at 0.05. The clinical and laboratory parameters of all patients at admission were obtained to find the independent risk factors for CS their hospital stay. Ultimately, SBP, DBP, TG, creatinine, globulin, LVEF, and CAG emerged as independent risk factors, which were further used to construct a nomogram.

The incidence of CS was 3.71% in this study. Previous studies have shown that timely revascularization has reduced the proportion of AMI complicated by CS to 5–7% in ST-elevation myocardial infarction and 2–4% in non-ST-elevation myocardial infarction. Given that this study cohort included both STEMI and non-STEMI patients, the observed incidence of CS falls within the expected range [13]. Currently, established risk stratification tools, such as the cardiogenic shock score, are primarily designed to assess mortality risk after CS has occurred [14]. However, there is a lack of models that can predict the onset of CS in AMI patients before hemodynamic collapse. Identification of high-risk individuals is clinically crucial, as it enables timely initiation of targeted interventions within the “golden window”, thereby significantly reducing short-term mortality and improving outcomes.

This study demonstrated lower SBP and DBP at admission in the case group, with the impact of SBP higher than that of DBP, making DBP a protective factor in the multivariate analysis. It is well known that during the early stages of shock, compensatory vasoconstriction occurs to maintain tissue perfusion, often resulting in elevated blood pressure [15]. Blood pressure fluctuations hold significant value for the diagnosis and prediction of CS. Close hemodynamic monitoring is therefore warranted in critically ill patients to prevent adverse events. CAG plays a central role in the management of AMI patients [16]. Early coronary angiography followed by prompt revascularization is widely recognized as the most effective strategy for limiting myocardial damage and preventing hemodynamic deterioration. The findings of this study also provided preliminary evidence that failure to perform CAG in the early stage is one of the risk factors for CS in patients with AMI.

In addition to blood pressure, elevated creatinine levels have also been identified as a risk factor. Creatinine levels serve as a diagnostic criterion for renal insufficiency, and elevated serum creatinine levels indicate impaired kidney function [17]. Chronic kidney disease leads to activation of the renin–angiotensin–aldosterone system, and this activation results in sodium and water retention, thereby increasing cardiac afterload [18]. The increased afterload further compromises already impaired cardiac function, reduces cardiac output, and markedly elevates the risk of shock. Moreover, renin–angiotensin–aldosterone system activation contributes to inflammatory processes by increasing IL-6 levels, which accelerates atherosclerosis progression, impairs coronary microcirculatory function, diminishes the heart’s compensatory capacity during acute ischemic events, and further heightens the risk of shock [19].

In contrast, protein-related biomarkers often exhibit a decreasing trend during CS. The systemic inflammatory response and endothelial dysfunction associated with CS cause substantial transcapillary leakage of plasma proteins into the interstitial space [20]. Combined with impaired hepatic synthesis and hemodilution, this results in markedly reduced serum protein levels. Hypoproteinemia disrupts plasma oncotic pressure, aggravates tissue edema, and contributes to abnormal blood volume distribution. As a negative acute-phase reactant, low protein levels also reflect heightened physiological stress and malnutrition. Evidence suggests that low protein is an independent predictor of poor outcomes in AMI patients with CS. Similarly, serum globulin levels, which encompass immunoglobulins and other proteins, may decline during critical illness due to impaired immune responses and overall protein depletion. One study found that reduced globulin levels in AMI patients were associated with an increased risk of long-term mortality, suggesting the prognostic value of these biomarkers [21]. Decreased globulin levels may impair antioxidative and anti-inflammatory functions, thereby contributing to inadequate myocardial healing and increasing susceptibility to multi-organ dysfunction. AMI patients exhibit reduced serum protein levels, which likely reflect a combination of capillary leakage, malnutrition, and immune dysregulation. Together, these alterations indicate a state of heightened physiological vulnerability, which is independently associated with hemodynamic instability and adverse outcomes.

Lipid profiles also exhibit distinctive alterations during CS. Although hypertriglyceridemia is widely recognized as a long-term risk predictor for cardiovascular disease, this study found that reduced TG levels constitute a risk factor for CS [22]. This may occur because severe physiological stress—such as inflammatory storms, neuroendocrine activation, and oxidative stress—can lead to decreased appetite and metabolic disturbances in patients, thereby causing a drop in TG levels [23,24]. Furthermore, hepatic synthesis of very low-density lipoprotein, which primarily transports TG, may be compromised during CS alongside decreased apolipoprotein B-100 levels, leading to reduced TG production [25]. Therefore, the characteristic lipid profile observed in patients with AMI complicated by CS is characterized by decreased TG, which likely reflects acute hepatic dysfunction and metabolic stress. This inverse change in lipid components suggests a state of severe metabolic derangement and carries prognostic significance.

In addition to the aforementioned metabolic indicators, impairment of cardiac function itself is equally an important dimension for assessing the risk of CS occurrence. LVEF serves as the core indicator for measuring left ventricular function and is strongly associated with clinical outcomes in AMI patients. Evidence from recent studies indicates that patients with mid-range LVEF (41–49%) and those with reduced LVEF (≤40%) exhibit comparable 30-day all-cause mortality rates, both of which are significantly higher than in patients with preserved LVEF (≥50%) [26]. This suggests that even moderate impairment in LVEF may confer substantial risk, highlighting the prognostic importance of LVEF as a stratification tool in AMI management.

According to the latest consensus, early revascularization of the culprit artery is recommended for patients with acute coronary syndrome complicated by CS [27]. For patients experiencing persistent low cardiac output and inadequate organ perfusion, mechanical circulatory support devices, such as intra-aortic balloon pump, Impella, or extracorporeal membrane oxygenation, should be considered. However, AMI patients often experience delayed diagnosis of CS due to atypical clinical presentations, the absence of standardized early recognition protocols, and the lack of invasive hemodynamic monitoring equipment in most medical institutions. This delay frequently leads to irreversible organ hypoperfusion and multiple organ dysfunction, thereby reducing the efficacy of pharmacological treatment and mechanical ventilation interventions [28]. Therefore, future research should focus on standardizing early recognition protocols, promoting rapid bedside biomarker testing, and leveraging remote monitoring technologies to ensure timely diagnosis and treatment for high-risk AMI patients, thereby reducing the incidence of CS and improving outcomes.

This study has some limitations. First, this is a retrospective case–control study, which may limit the accuracy and completeness of data collection due to reliance on existing medical records. Second, some potential confounding factors were not available and therefore could not be adjusted for in the analysis, which introduces some residual bias and imbalance. Finally, the findings are derived from a single-center cohort and should be interpreted with caution. External validation in balanced cohorts is required through larger, prospective, multi-center studies.

## 5. Conclusions

This retrospective case–control study identified SBP, DBP, TG, creatinine, globulin, LVEF, and CAG as risk factors for the development of CS in patients with AMI. A nomogram based on these seven variables provided a practical tool for estimating the risk of CS in AMI patients and may assist clinicians in early risk stratification and management.

## Figures and Tables

**Table 1:** Baseline characteristics before and after PSM between the two groups.

Characteristics	Before PSM (*n* = 10,084)				After PSM (*n* = 748)	
	Case Group	Control Group	*p* Value	Case Group	Control Group	*p* Value
	*n* = 374	*n* = 9710		*n* = 374	*n* = 374	
Male (*n*, %)	272 (72.7)	7903 (81.4)	<0.001	272 (72.7)	274 (73.3)	0.869
Age (years)	63 (55, 71)	61 (52, 69)	<0.001	63 ± 12	63 ± 11	0.954
STEMI (*n*, %)	263 (70.3)	5600 (57.7)	<0.001	263 (70.3)	264 (70.6)	0.936

PSM, propensity score matching; STEMI, ST-elevation myocardial infarction.

**Table 2:** Baseline characteristics between the two groups.

Characteristics	Total	Case Group	Control Group	*p*-Value
	*n* = 748	*n* = 374	*n* = 374	
**Demographic Characteristics**				
BMI (kg/m^2^)	24.13 ± 3.14	23.73 ± 3.23	24.52 ± 2.99	0.001
Smoking, *n* (%)	223 (29.8)	111 (29.7)	112 (29.9)	0.936
Drinking, *n* (%)	95 (12.7)	39 (10.4)	56 (15.0)	0.062
Recurrent AMI, *n* (%)	45 (6.0)	25 (6.7)	20 (5.3)	0.442
**Medical History, *n* (%)**				
Hypertension	344 (46.0)	141 (37.7)	203 (54.3)	<0.001
Diabetes	232 (31.0)	102 (27.3)	130 (34.8)	0.027
**Vital signs**				
Heart rates (beats/min)	75 (65, 86)	76 (63, 89)	75 (67, 84.50)	0.917
SBP (mmHg)	103 (89, 129)	89 (84, 90)	126 (111, 140)	<0.001
DBP (mmHg)	67 (58, 80)	59 (54, 65)	77 (68, 88)	<0.001
**Biochemical Parameters**				
Hb (g/L)	136 (123, 148)	134 (120, 147)	139 (126, 150)	0.001
WBC (10^9^/L)	9.59 (7.18, 12.18)	10.47 (7.80, 13.16)	8.76 (6.82, 11.16)	<0.001
NEU (10^9^/L)	7.56 (5.15, 10.37)	8.34 (5.60, 11.31)	6.56 (4.81, 9.22)	<0.001
NEU% (%)	79.10 (71.32, 86.10)	81.05 (72.90, 86.70)	77.10 (69.05, 84.33)	<0.001
LYMPHO (10^9^/L)	1.33 (0.93, 1.85)	1.37 (0.92, 1.89)	1.30 (0.95, 1.80)	0.498
LYMPHO% (%)	14.62 (9.46, 21.54)	13.45 (8.85, 19.89)	15.70 (10.06, 23.52)	0.002
CRP (mg/L)	55.05 (27.49, 84.90)	58.78 (31.90, 85.84)	49.63 (21.43, 83.46)	0.036
HbA1c (%)	5.90 (5.00, 7.00)	5.80 (5.50, 6.50)	6.10 (5.60, 7.50)	<0.001
LDL (mmol/L)	2.17 (1.64, 2.82)	2.16 (1.60, 2.84)	2.20 (1.68, 2.78)	0.437
HDL (mmol/L)	0.93 (0.80, 1.07)	0.93 (0.82, 1.08)	0.93 (0.79, 1.07)	0.526
TC (mmol/L)	3.88 (3.23, 4.62)	3.79 (3.15, 4.57)	3.95 (3.29, 4.69)	0.091
TG (mmol/L)	1.18 (0.82, 1.69)	1.08 (0.73, 1.53)	1.27 (0.90, 1.80)	<0.001
hs-cTnT (ng/dL)	0.56 (0.11, 1.99)	0.60 (0.13, 2.37)	0.54 (0.10, 1.61)	0.161
hs-cTnI (ng/dL)	1590.55 (239.33, 9741.61)	2580.73 (295.36, 18,365.53)	1194.84 (227.67, 6812.40)	0.260
LDH (U/L)	320.00 (231.00, 554.00)	350.00 (240.50, 655.75)	292.50 (228.00, 439.25)	<0.001
CK (U/L)	387.00 (119.09, 1236.50)	587.14 (145.50, 1460.00)	301.00 (107.75, 901.00)	<0.001
CK-MB (U/L)	45.65 (18.00, 147.75)	63.40 (20.04, 176.63)	33.00 (16.00, 109.93)	<0.001
NT-proBNP (pg/mL)	1001.85 (326.35, 2973.38)	1372.50 (330.25, 3553.65)	813.10 (315.18, 2295.75)	0.002
Urea (mmol/L)	5.91 (4.76, 7.56)	6.16 (4.94, 7.85)	5.57 (4.55, 7.10)	<0.001
Creatinine (µmol/L)	67.00 (55.00, 85.00)	72.00 (57.00, 96.00)	64.50 (53.00, 79.25)	<0.001
Globulin (g/L)	25.43 ± 4.33	24.76 ± 4.47	26.10 ± 4.08	<0.001
Albumin (g/L)	36.97 ± 5.06	36.30 ± 5.12	37.65 ± 4.91	<0.001
AST (U/L)	61.00 (29.00, 170.00)	84.00 (32.00, 240.00)	49.00 (27.00, 124.50)	<0.001
ALT (U/L)	37.00 (22.00, 63.00)	41.50 (24.75, 72.50)	32.00 (21.00, 58.50)	<0.001
D-dimer (mg/L)	0.68 (0.38, 1.52)	0.83 (0.46, 2.32)	0.55 (0.33, 1.01)	<0.001
FDP (mg/L)	2.22 (1.30, 5.41)	2.50 (1.50, 7.55)	2.00 (1.20, 3.60)	<0.001
**Echocardiology**				
LVEF (%)	51 ± 11	49 ± 11	54 ± 11	<0.001
Aortic regurgitation, *n* (%)	17 (2.3)	6 (1.6)	11 (2.9)	0.220
Mitral regurgitation, *n* (%)	89 (11.9)	45 (12.0)	44 (11.8)	0.910
**Treatment**				
PCI, *n* (%)	621 (83.0)	303 (81.0)	318 (85.0)	0.144
CAG, *n* (%)	687 (91.8)	330 (88.2)	357 (95.5)	<0.001
**Clinical outcomes**				
In-hospital stay (days)	4 (3, 7)	5 (3, 7)	4 (2, 6)	0.009
In-hospital mortality (%)	26 (3.5)	20 (5.3)	6 (1.6)	0.005

BMI, body mass index; SBP, systolic blood pressure; DBP, diastolic blood pressure; Hb, hemoglobin; WBC, white blood cells; NEU, neutrophils; NEU%, neutrophil percentage; LYMPHO, lymphocytes; LYMPHO%, lymphocytes percentage; CRP, C-reactive protein; LDL, low-density lipoprotein; HDL, high-density lipoprotein; TC, total cholesterol; TG, triglycerides; hs-cTnT, high-sensitivity cardiac troponin T; hs-cTnI, high-sensitivity cardiac troponin I; LDH, lactate dehydrogenase; CK, creatine kinase; CK-MB, creatine kinase-MB isoenzyme; NT-proBNP, N-terminal pro-brain natriuretic peptide; AST, aspartate aminotransferase; ALT, alanine aminotransferase; FDP, fibrin degradation products; LVEF, left ventricular ejection fraction; PCI, percutaneous coronary intervention; CAG, coronary angiography.

**Table 3:** LASSO-logistic and multivariate logistic regression between the two groups.

Characteristics	LASSO-Logistic Regression		Multivariate Model	
	Assignment	Coefficient	OR (95%CI)	*p*-Value
BMI	Continuous variable			
Drinking	Yes = 1, No = 0	−0.1438	0.636 (0.309, 1.310)	0.220
Hypertension	Yes = 1, No = 0	−0.1433	1.062 (0.634, 1.781)	0.819
Diabetes	Yes = 1, No = 0			
SBP	Continuous variable	−4.2030	0.866 (0.844, 0.888)	<0.001
DBP	Continuous variable	0.6490	1.031 (1.001, 1.063)	0.046
Hb	Continuous variable			
WBC	Continuous variable			
NEU	Continuous variable	0.3581	1.059 (0.978, 1.146)	0.161
NEU%	Continuous variable			
LYMPHO%	Continuous variable	0.5165	1.026 (0.989, 1.064)	0.166
CRP	Continuous variable	0.2092	1.005 (0.999, 1.011)	0.119
HbA1c	Continuous variable	−0.3994	0.877 (0.730, 1.054)	0.161
TC	Continuous variable	0.2540	1.273 (0.960, 1.688)	0.093
TG	Continuous variable	−0.5480	0.561 (0.385, 0.820)	0.003
LDH	Continuous variable			
CK	Continuous variable			
CK-MB	Continuous variable			
NT-ProBNP	Continuous variable			
Urea	Continuous variable			
Creatinine	Continuous variable	0.2631	1.005 (1.000, 1.010)	0.048
Globulin	Continuous variable	−0.3917	0.915 (0.862, 0.972)	0.004
Albumin	Continuous variable	0.1642	1.034 (0.976, 1.095)	0.256
AST	Continuous variable			
ALT	Continuous variable	0.8541	1.002 (0.999, 1.004)	0.050
D-dimer	Continuous variable	0.1874	1.027 (0.985, 1.071)	0.206
FDP	Continuous variable			
LVEF	Continuous variable	−0.7371	0.951 (0.928,0.975)	<0.001
CAG	Yes = 1, No = 0	−0.3858	0.183 (0.058, 0.574)	0.004

LASSO, least absolute shrinkage and selection operator; OR, odds ratio; CI, confidence interval; other abbreviations as in Table 2.

## Data Availability

The data underlying this article cannot be shared publicly due to the privacy of individuals that participated in the study. The data will be shared upon reasonable request to the corresponding author.

## Abbreviations

The following abbreviations are used in this manuscript:

AMI	Acute myocardial infarction
CS	Cardiogenic shock
PSM	Propensity score matching
OR	Odds ratio
CI	Confidence interval
AUC	Area under the curve
LASSO	Least absolute shrinkage and selection operator
LVEF	Left ventricular ejection fraction
CAG	Coronary angiography
TG	Triglycerides
SBP	Systolic blood pressure
DBP	Diastolic blood pressure
